# Professional Ideals and Practical Patients: A Problem in Practice

**Published:** 1915-12

**Authors:** E. Harrison


### PROFESSIONAL IDEALS AND PRACTICAL PATIENTS: A PROBLEM IN PRACTICE.

BY E. HARRISON, L.D.S.

---

In most pursuits in life there are two great channels along which man's efforts are directed. On the one hand, there is art in some form or another—a production of the individual worker, which can not be communicated to another in terms of mathematical exactitude, but which is a part of the individuality. Amongst the finished products of art, we may recognize the characteristics of a given worker, but we can not reproduce them at will. There may perhaps be a more or less accepted standard or fashion to which all art conforms, but in its ultimate end all art is the expression of the individual.

As a contrast there is science, with its standards in every department, which must be observed with undeviating accuracy if one wishes to acquire a reputation for really scientific work. The work of a scientist must be capable of proof by other workers, because all proceed along the same path, and each is armed with the self-same foot rule, by which to test the position of the original worker. Art may be influenced by instinct, temperament or environment; but science can not be swayed a hair's breadth by any of these forces. Everything must conform rigidly to the chemical test, or definite standard, if science is to be satisfied.

From personal observation, as well as from generally expressed opinion, I should say that it is comparatively rare to find these two qualifications in one and the same person, to any advanced degree. As a general rule, a man who has a capacity for art in any form finds it exceedingly irksome to be trammelled by the rigid rules and regulations which of necessity govern the pursuit of science, so that one might almost formulate a dictum that attainment of any outstanding degree of excellence in an art almost precludes the possibility of a similar proficiency in science.

And yet dentistry is essentially of a hybrid construction, because it makes demands on both science and art to a considerable degree. A case in point which occurred to the writer a few years ago will serve as an illustration.

A patient presented himself, expressing dissatisfaction with a complete upper denture she was then wearing, stating that her entire expression was altered and asking if nothing could be done to restore something of the original contour. Upon examination, I will undertake to say that if I had passed the case she was then wearing round this room, there would not have been two opinions as to its excellence and beautiful work. Everything was as perfect as one could wish for, but there was one blemish which was not observable until the case was in the mouth. It had no artificial gum, *i. e.*, the denture was mounted to the natural gum at the front, and though the articulation was perfect, the effect was almost as bad as without the plate altogether. In other words, science was predominant—in fact perfect, but art was almost absent.

Well now. Here we have the professional ideal. There is to be art in the planning of the work, art in the arrangement of the individual teeth, art in contouring, but science steps in and lays down the laws which must govern all, even artistic work, if it is to be useful and enduring in the mouth.

I suppose if one were to state the highest ideal of dentistry, it would be to preserve every tooth of every individual, or at least to build up any missing portions, so that we might shake off this mortal coil with the natural teeth in position, having been free from dental pain and possessed of perfect mastication all our lives.

A quaint old artisan whom I knew was ever wont to admonish his boys thus: "Aim at the moon, my boy.

You may reach the top of a tree.'' And though the ideal I have sketched may be regarded as Utopian, it is nevertheless as well to bear it constantly in mind, for advances in scientific attainment, just as wonderful, have been actually accomplished of late years in other domains.

But alas! I am afraid most of us have descended from the celestial sphere of our student days, to a more terrestial habitation of actual accomplishment. Daily contact with the profound carelessness of the average patient, and the deep-rooted idea, which has not even yet been eradicated from the minds of the masses, that a dentist exists only for the purpose of ''yanking out'' an aching tooth, has been largely responsible for this descent, and it is generally a matter of making the best of a bad job. And it is right that it should be so, always supposing that we do not allow the strong growth of material considerations to entirely choke those ideals, which are of such tender growth that, unless sedulously cultivated they are prone to wither and die.

I am not for a moment insinuating that ideals are never to be departed from. I believe there are occasions when it is desirable, and even necessary, to do so. But these occasions are rare, and may perhaps be regarded as the exceptions that prove the rule. For instance, the whole is greater than a part, and therefore the general health of the patient must be considered as being greater than the preservation of the natural teeth. I will put it before you in a concrete form, so that I may make my meaning perfectly clear.

More and more, as the necessity for dental treatment becomes recognized by the masses, we are called upon to treat the teeth of young children. And though this is what we would wish, it has added a heavy burden and caused an even greater than normal strain on the patience and vitality of the dentist. The little patients, for some reason or other, are often regaled with lurid stories of the brutality of the dentist, for some days before their visit, and it would not be difficult for even one unacquainted practically with such scenes to imagine the effects of such tales on the highly imaginative child mind, especially in these days of over-developed nervous systems and hysterical patients.

A child of this type, who was brought to the writer a few months ago with a large cavity and exposed pulp, permitted the insertion of a dressing without demur. But on the second visit a week later, though the pulp was dead and no surrounding tenderness present, she screamed from the moment she sat upon the chair until she vacated it. The mother suggested holding the patient down, but I opined that by the time the operation was finished, had I persevered, the child would have been partially demented by fright. So I advised the mother to bring the child again if she thought there was any reasonable hope of her seeing facts in their proper perspective—if not there was nothing but extraction.

The whole is greater than a part; and the general health of the patient must, in my opinion, always be paramount over the preservation of the natural teeth, when these two things happen to clash.

I suppose in dentistry as in other professions, the possession of years of experience consists very largely in knowing "when not to do." Coleridge defined wisdom as "common sense in an uncommon degree," and this should be a pre-eminent attribute of the dental surgeon. As a student his time is taken up in learning what to do in given contingencies, but when he has passed the portal of efficiency and received the hall-mark of the examining authorities, his life is very largely made up in learning when to depart from these general lines for special reasons.

I have stated previously what I consider should be the highest ideal of dentistry, but as that would appear under present conditions and in the state of our present-day enlightenment, impossible of attainment, we must bend all our efforts to approaching as near as possible to our ideal from the material with which we are presented. Success is generally a compromise between the best and the worst, or to put it another way, we must see to it that the finished result bears the utmost likeness to our ideal, that the original conditions will by any possibility permit. One of the fascinations of the profession indeed, is, I think, the diversified problems which have to be overcome. It is no less than wonderful when we consider the matter that so many differentiated conditions can possibly exist in the oral cavity, though (speaking roughly) all mouths follow the same pattern, and the teeth are similar in pattern, position and relationship. But this is a commonplace.

There are, however, certain broad lines which I think we should observe in every case. We should place first things first; and under this heading, I take it, our first object should be to make the mouth perfectly healthy. Whatever use an artificial substitute may be, in the matter of appearance, or comfort, or assistance in mastication, it is not fulfilling its primary function unless it is first of all and above everything—healthy. I had almost said —I think I will even now commit myself to the opinion— that I would prefer to dismiss a case with slightly imperfect mastication and perfect health rather than with perfect mastication, but in an unhealthy condition, if I were presented with one alternative only. Of course, I am not insinuating that we should not use our utmost endeavours to ensure perfect mastication and a pleasing, natural appearance, but as I said before, first principles must be placed first. We are witnessing on every hand the pernicious effects of the flora of unhealthy mouths, on the alimentary tract, and if our treatment tends to perpetuate or increase existing unhealthiness, then we can not complain if the medical profession steps in and orders the removal of perhaps a choice piece of work in the form of a bridge. I do not say we are in the habit of doing such things, but do we always consider health first and other attributes afterwards?

And here I would modestly venture to express an opinion, that we are well advised under existing conditions of dental practice not to make a particular hobby of one department of dentistry, to the exclusion of all others. Unless the day comes (which is not at all unlikely) when we may have specialists in different branches of dental work, it seems to me that if one indulges in a particular style of treatment, to the exclusion of nearly all other types of work, occasions will certainly arise, sooner or later, when a case presents which may, with very serious difficulty and great ingenuity, be treated by the favourite method with only very indifferent success; whilst if treated by a simpler, and perhaps more obvious method, the success would have been much greater and the time and ingenuity immeasurably less.

As a case in point, one might instance a bridge which the writer recently saw. Owing to the position of the teeth and for other reasons, the case presented many obstacles in the way of making a bridge satisfactorily, and a plate would have offered a much more satisfactory (and certainly much easier) solution of the problem, but the bridge had been inserted, with the result that it had to be removed—roots as well—a short time after. Naturally, the presentation of unusual difficulties to one's pet method only stimulates one's energy and initiative to overcome them, but one should always allow sober judgment to occupy the supreme court of appeals as to what is the best method to adopt in any given set of circumstances.

I am not offering this as an excuse for lack of proficiency in any department whatever. If we had dental specialists it would be easy to refer a case of unusual difficulty to, say, an orthodontist or other specialist. One has, however, seen cases where—referring to a previous remark—if the operator's eyes have been turned towards the heaven of his ideals, his hands appeared at the same time to be earthwards in the direction of his patient's pocket.

The idea works out in a number of ways. The highest interest of a patient is not served by inserting a bridge where a plate would be better and *vice versa,* and one is not justified in inserting an inlay if a gold filling would better fulfill the required conditions.

How necessary it is for us to inculcate in our patients the habit of regular and systematic visits for the purpose of inspection. We are constantly complaining of the ignorance and carelessness of the masses in regard to their teeth, but do we seize every opportunity to impress this necessity on our patients? I have found it was a great help to me to inform my patients, say, when I had finished all attention necessary to restore a number of defective teeth, and made the mouth as healthy as possible, that I did not charge any fee for examination if the patient would come regularly for inspection. I do not know what is the practice of my colleagues, but I find it has been a help, and worked well for some years. I generally say on dismissing a patient, ''You will be all right for perhaps three months. If you come then you may not need any attention; but if you delay, a single cavity may mean half a dozen in a short time.''

But it sometimes happens that one reaches a point in conservative work where a very nice problem obtrudes itself rather prominently. Say a patient has been under more or less continuous observation and treatment since childhood. The patient has attended as often as requested and you have inserted filling after filling, faithfully doing your duty with every possible care. You have thought, perhaps, that after a few years this persistent literal and metaphorical ''rot'' would cease and be replaced by a cycle of healthier and more resistant years. It may even well be that up to this point not a single natural tooth has had to be sacrificed.

But there comes a time when it is impossible to disguise the fact that conservative treatment is no longer of any avail whatever. One has perhaps hoped to retain the lower incisors and canines, at least, but at last even these are attacked by the all too-well-known white decay, at the approximal surfaces. Molars and bicuspids are nearly all filling, and where a fresh cavity is noticed it has travelled towards the old filling, and perhaps the latter is loose and worse than useless.

What is to be done? The patient is perhaps of a practical turn and not always inclined to accept without demur the professional man's advice. He wants to know what is ahead. He has followed the advice tendered to him so far religiously. He has attended dozens of times. Occasionally—happy memory—he has been told that his mouth is perfectly healthy and requires no attention at the dentist's hands for a few months. On other occasions—and these constitute by far the largest majority—he has undergone the inconvenience and pain of filling with all the other delicate little attentions of the modern dental practitioner. What is to happen if he accepts the further advice now offered to him and resorts to crowns or bridges? And indeed ought one conscientiously to recommend a treatment of that sort under the conditions I have sketched? If he has the conservative work performed, can the dentist give him any reasonable assurance that the crowns will last for any reasonable period? The cost is likely to be considerably more than a plate carrying the same number of teeth. And on the other side there is the certainty that the absorption following extraction will make a difference in the appearance. But—given the fact that the absorption has progressed to a certain point after extraction—there is the certainty that the plates will be useful and satisfactory for a number of years. And in any event there is the probability that the patient will have to come to the denture in the end, even if the system of crowns and bridges is adopted in the meantime.

I want, if possible, to indicate in a few words the essential characteristics of a mouth which is often associated with a certain class of patients. There is, perhaps, not a perfectly sound tooth in the whole mouth, and most of the molars and bicuspids are in a very broken-down condition. It might, perhaps, be possible with infinite trouble to find sufficiently strong and healthy walls in a few of them to hold fillings in position. Many of the others it may well be could be crowned if such a course was deemed desirable. Some would be absolutely unsavable and only extraction would be possible for these. But there would be others—perhaps the lower incisors and canines—which obviously were the best, or rather the least decayed, of any in the mouth. But even these, unfortunately, do not give any great promise of permanency, even if perfectly filled and restored. The white decay in the approximal surfaces, and the general appearance of the tooth structure, all seem to indicate that the teeth are not very resistant, and would be likely to become attacked by caries in other places, even if filled for the time being. Add to this a general impression, which may sometimes be gathered from the conditions presented, that for some years the mouth had undergone a process of slow decay, but for a few months before the consultation a more rapid species of caries had attacked the teeth and, to use a commercial phrase, had caused a sudden slump.

The cavities in the front teeth may not be extensive; it is the character of the decay that makes one apprehensive of the possibility of saving them. I grant you that there are many patients who would scarcely give the dentist cause for a moment's consideration. Unlimited time at a patient's disposal and the possession of sufficient means and a disposition to have "absolutely the best,"

coupled with a more or less Spartan temperament, would undoubtedly prompt the professional man to advise a strictly conservative treatment, and if in the course of a year or so some other method had to be adopted—well! the patient has the satisfaction of knowing that nature has had every possible chance, and it is only when the absolute limit of conservative work has been passed—or the limit of the patient's patience—that one would resort to extraction.

But we are not considering that case at the moment. There are always numbers of people who are very willing to pay high fees for "the very latest" treatment, and even if it is unsuccessful they have the satisfaction (barren though it may be) of knowing that they have tried it. But our typical case is not of that species, and I think I have made it sufficiently clear to indicate what I mean.

The question I want to ask is, are we justified in advising any kind of conservative treatment to a patient in such a case? We are advised on the authority of a very eminent medical practitioner to give our diagnosis (or treatment), but never our reasons. I am not sure that I altogether agree with that advice in every case, excellent as it is in the main. Perhaps the same authority would agree that there is a difference between the practice of medicine and dental surgery. The least we can do, however, in the case under consideration is to tell the patient the relative advantages of the two courses, and their disadvantages. I say this because I believe there is a feeling in every dentist's mind that he takes no pleasure in the slow and tedious process of building up or producing anything that is not likely to be enduring or to serve its full purpose.

I think this feeling is common in any good workman, to whatever trade or profession he may belong. And I do not think I am picturing the profession as more altruistic than it actually is, when I say that every practitioner likes to know and likes the patient to appreciate too—though he may not go out of his way to enlighten him—that he has received value for the fees he has paid.

There is also that eminently practical patient, who comes to you with a tooth very badly decayed, and you do your best to restore and preserve for some time longer the neglected member. The filling gives way in a few months' time—perhaps a thing you had foreseen at the time it was inserted—and the patient turns up immediately, with the air of an injured innocent, and all but demands you to refill it without fee.

A certain section of the public seems to demand that if anything goes wrong with the dentist's handiwork, he is in duty bound to make it right without fee, but they would never dream of demanding a return of the fees paid to a medical man, even if the patient had been treated for twelve months without improvement.

I think it is high time we had some common understanding among ourselves as to the position we should take in cases of that sort. There is no doubt whatever in my own mind that the dentist charges his fees for whatever skill he may be able to put into the services he can render. Study, training and natural aptitude, coupled with experience, will undoubtedly make a difference in the respective capacities of various professional men, but it is absurd to suppose that a dentist is to be paid by results. I remember one case, indeed, where the two centrals were the only teeth left in the maxilla, and both these were suffering seriously from caries. I advised the patient to have them removed, but she declined, and I pointed out that even if they were filled they would probably not last two years. She, however, insisted on retaining them, and after further impressing on her the fact that an entirely new denture would be necessary, after the centrals were removed, I weakly gave way.

A little more than two years afterwards that same patient returned minus the two front teeth, and on again explaining that a new denture would be necessary, she roundly abused me, and told me I ought not to have done such work if I knew the teeth would not last. But I am afraid the patient found me in a less complaisant mood on this occasion, and she came back ultimately to what I wished.

I have chosen this subject, and written as I have, largely in the hope of being of service to some of our younger men. I trust the older practitioners will not find it entirely without interest.—*Dental Record.*

